# Experience of group conversations in rehabilitation medicine: methodological approach and pilot study

**DOI:** 10.1007/s12682-015-0208-7

**Published:** 2015-05-07

**Authors:** Cecilia Perin, Massimiliano Beghi, Cesare Giuseppe Cerri, Federica Peroni, Barbara Viganò, Cesare Maria Cornaggia

**Affiliations:** Department of Surgery and Translational Medicine, University of Milano Bicocca, Via Cadore 48, 20052 Monza, Italy; Department of Mental Health, “G.Salvini” Hospital, Via Forlanini 121, Garbagnate Milanese, Milan, Italy; Cognitive Neurorehabilitation, “Zucchi” Clinical Institute, P.Za Madonnina 1, Carate Brianza, Italy

**Keywords:** Rehabilitation, Conversation analysis, Outcome, Trauma, Stroke, Pilot study

## Abstract

The restoring of equilibrium after a traumatic event makes it possible to give a new significance to patients’ existence, and healthcare professionals simultaneously find themselves very close to questions of pain and disability. For these reasons, we introduced weekly group meetings of healthcare professionals and patients suffering from vascular, traumatic or neurological accidents, and meetings of professionals only at the Neurocognitive Rehabilitation Day Hospital of the University of Milan Bicocca. The aim of this paper is to identify possible indicators of changes in patients’ existence through a conversational analysis, describing the experience at the light of methodological approach and reporting the results of a pilot observational study. The patient meetings began in October 2011 and led to a process of greater closeness and trust that was expressed by means of words, gestures, emotional participation, and non-verbal communication. The pilot considers the evolution of indicators in a sample of 14 patients for a period of 9 months and a timeframe of 3 months. Supportive interventions decreased while elements of sharing progressively increased, leading to progressive increased consciousness of both self and the disease. The group of professionals found that being together allowed them to distinguish performance as the use of their technical skills from understanding the other and his/her experience as part of their own, and not only linked to the disease. The professionals’ reflections on their experiences led to the emergence of two possible ways of looking at a patient: as somebody other than me or somebody other like me.

## Introduction

Anyone having to face a traumatic event (whether it is due to a stroke or an accident at work or in the street) has to employ a series of strategies aimed at restoring an equilibrium that makes it possible to accept and give new significance to his or her future life.

A traumatic event is an “eruption of vehement emotions that it make it impossible to use mental plans to give significance or face the event insofar as it is unforeseen and cannot be cataloged on the basis of consolidated patterns of meaning and interaction” [1].

When they are at the patient’s bedside, healthcare professionals (physicians, nurses, physiotherapists, speech therapists, etc.) find themselves at extraordinarily intensive close quarters not only with the pain of the other, but also with the harshness of an experience whose centrality lies in a sense of impotence.

Various studies have found that a trauma is initially followed by a high prevalence of psychiatric (particularly depressive, anxiety and adjustment) disorders [2], and these seem more related to the existence of the trauma itself than to the type and severity of trauma. Furthermore, these psychiatric disorders usually last for a long time after the event and often affect the prognosis of rehabilitation, and therefore the efficacy of the undertaken interventions [3].

On the other hand, healthcare professionals have to face disability more closely than ever before, and they have built impregnable defensive barriers often concealed by an excessive emphasis on their technical competences; this can increase a risk of allowing an over-detachment from the anguish circulating the environment surrounding their patients.

This mechanism reveals the activation of a natural defensive system that makes it possible to face situations of pain and death without becoming fragmented. Lazarus and Folkman [4] define this as “coping” which they divided into the two categories of emotion-focused and problem-focused coping, both of which are characterized by making every cognitive and behavioral effort to act in the face of a potentially stress-inducing situation. Problem-focused coping relies on using existing competences and skills to eliminate the cause of the stress. Although the possession of technical skills makes it possible to face and give significance to a painful experience, it may in extreme cases lead to emotive avoidance and the use of technical automatisms that exclude the emotional sphere.

The question then becomes whether it is possible to adopt tools that can help the circulation of both rational and emotional contents in such a way as to re-introduce the deepest parts of oneself, thus making it possible to face a difficult and traumatic reality with an awareness of the limitations it implies while simultaneously rediscovering life pleasures.

Especially in patients with cognitive impairment, deficits in these skills are common. These patients tend to lose the ability to communicate their thoughts and needs, and to interact socially and sustain personal relationships with others; for these reasons, patients become frustrated at their loss of self-expression, and there is a strong link between impaired communication and growing behavioral concerns [5]. As has been shown in patients with Alzheimer disease, the capacity to treat or reduce the progression of communication deficits would prolong patient independence and have a deep impact on the patients’ and caregivers’ quality of life [5, 6].

The aims of the experience will thus be the following:

1. For the patients: to reactivate their skills remaining after their vascular, traumatic or neurological accidents.
2. For the professionals: to elaborate the emotive charge related to the situations they have had to face in their job.

For these reasons, in the Neurocognitive Rehabilitation Day Hospital (Director: CP) we introduced group conversations organized as follows: meetings of a group of healthcare professionals and patients followed by meetings of a group of professionals only. This paper describes and comments on these meetings two years after they began and discusses the theoretical approach as well as the pilot study results.

## Materials and methods

### Context

The meetings took place at the Neurocognitive Rehabilitation Day Hospital of the Zucchi clinical Universitary Institute (Italy) in a room in which the patients and professionals sat in the same circle so that they could see each other to build a symmetrical relationship that was not characterized by differences in role. Every meeting lasted 1 h and was followed by a 10- to 15-min professionals’ reflection meeting. Our study provides an observational design of the experience and presents the results of the pilot study on 9 months of observation.

### Group of patients

Each group meeting consisted of patients accessing the Neurocognitive Rehabilitation Day Hospital. All of the patients required multidisciplinary treatments to rebuild their skills after their neurological accidents (e.g., vascular, traumatic, etc.). These have been joined by about ten professionals, including the psychiatrist who conducted the sessions (CMC), the nursing coordinator, the physiotherapists, a neuropsychologist (BV), a speech therapist (AS), a clinical psychologist (FP), and sometimes post-graduate students. The patients were heterogeneous in terms of pathology and severity.

The weekly meetings began in October 2011 and still continue, but in the pilot study we consider the first 9-month period (October 2011 till June 2012) only. The schedule was fixed to be able to integrate the meetings with the planning of other activities and to give the participants an element of rituality (which led to positive outcomes such as their starting to prepare themselves beforehand). Each meeting lasted 1 h and began with some general questions and reflections concerning the subjects discussed during the previous meeting. These prompts led to the creation of new associations and shared thoughts with the intervention of the group leader, who continuously reproposed the themes and tried to involve all of the participants. At the end, the leader needed to intervene less as a result of the trust and closeness of patients who often questioned each other and proposed subjects for discussion.

### Group of healthcare professionals

At the end of the patients’ group, the healthcare professionals meet with the following aims:

- Reflections on the conversation with patients.
- Pointing out on their emotional experiences.
- Reflecting on the existing connection between patient and healthcare professional about the event “trauma” in terms of shared experience as “human experience”.
- Listing the keywords risen in the conversation.

### Theoretical methodological assumptions

The group of patients was created on the model of Giampaolo Lai, the founder of conversationalism [7], which is based on the principle sustained by Lai that …“conversation” and “communication” are two very different functions. Communication is an interactive process of exchanging information that is mediated by any type of signal or symbol (visual, acoustic, gestural, linguistic) and governed by logical and pragmatic rules, whereas conversation is purely linguistic and consists of a sequence of discrete elements (the words said by one person in the presence of another) governed by grammatical rules; insofar as it is possible to have a “conversation without communication”. In the forefront and beyond the interest in research, there was a clinical scope to valorize the person (however deprived or apparently deprived) by restoring the own dignity of a human being with whom it is possible to hold a simple conversation.

The aim of conversationalism is to favor the access of a patient’s conversation toward possible worlds in which the conversant agrees to accompany the patient by picking up his or her words without question and attempting to build harmonies and intersections with them. Unlike the real world, the possible world is not ruled by consistency, logic and the principle of non-contradiction, but is constituted by the words that pass from world to the other [8].

The individual narrative motifs of each patient may be characterized by the presence of inconsistencies and the lack of any logical connection between them; however, if they are collected from one and another and then repeated (i.e., anaphorically restituted), they give rise to a circulation of unitary narrative motifs which, partially as a result of the cohesion of the text obtained, increase the cohesion of the group discourse regardless of the possible incoherence of the discourse of the individual patients.

Setting aside their difficulties of communicating and maintaining the conversational theme, the subjects communicate affectively and report a gratifying emotion as if there were a sort of “split” between verbal and affective communication.

Conversationalism [8–10] is based on putting a conversant and interlocutor together in a given space for a defined period of time, and then beginning the conversation in a manner that the conversant considers most appropriate to encourage the interlocutor to speak, to speak long enough, to speak happily, and to keep the conversation going without questioning the interlocutor, and without interrupting or completing what he or she is saying. The leader must simply try to return the narrative motif to the interlocutor (even by administering of fragments of his or her own autobiography), and above all, never make any interpretation.

In comparison with the holistic neuropsychological rehabilitation programs [11, 12] mainly centered on the individualized definition of objectives, the therapeutic decisions supported by the rehabilitation team, the rehabilitation of awareness and not only cognitive functions, and the ecology of the therapeutic setting, this approach seems to be freer of pre-ordained rules.

### Indicators

All of the conversations were video-recorded. Some interventions were taken as indicators and counted to verify whether there was any change in their frequency. These were identificative interventions (parts of what is said by the interlocutor are recognized as belonging to his/her personal experiential sphere), completing interventions (the completion of what the other says on the basis of personal experiences), supportive interventions (characterized by the capacity of non-judgemental empathic listening to the account heard) and interventions of sharing (introduced on the basis of a recognition of the same effort albeit starting from a different experiential origin).

Identificative interventions are observed when parts of the story of the other are recognized as belonging to the self. This is the first index of an affective communication insofar as it “de-latentises” what may be empathy toward something being said.

Supportive interventions are primarily characterized by non-judgemental, active and participative listening, and an ability to offer reflections and points of sharing based on an understanding of what the other is experiencing. The significant aspect is that these types of interventions highlight not only the cognitive capacity of attention, but also the affective capacity of recognizing the emotive state of the other. The interventions of sharing are based on the possibility of being with the other in living certain emotive experiences and the fatigue involved in participating in a rehabilitation program.

#### Respect for the rules

Respecting the rules of conversational courtesy (the principles underlying the logic of courtesy are not to impose, offer alternatives, put your interlocutor at ease), awaiting your turn (let your interlocutor finish what he or she wants to say before putting forward your point of view), and continuing to look at your interlocutor creates an ordered form of communication unhampered by overlapping voices. In addition to being related to respect for the other, it also underlines a willingness to listen so that what is said can represent an important opportunity for everyone to engage in self-reflection.

#### Non-verbal communication

Refusal, crying and smiling are the most common non-verbal manifestations observed during group interactions; they represent three important cornerstones of non-verbal affective communication and underline the participation of the different group members. However, the significant aspect is that these reactions were observed not only among the patients, but also among the professionals.

## Results

In this experience, the results are not always quantifiable since it is not possible to translate in numbers what happens in these groups but we will give the readers some indicators of the evolution of the group. Moreover, the composition of the group is flexible and changed in time. In the pilot study (see below), we choose the sample of the original conversation group.

### The experience

During the construction of the group of patients (the group that will be described in greatest detail in this paper), it was possible to see the development of a greater closeness and trust among the members, which was made explicit by means of words, gestures, emotional participation and non-verbal communication (see the analysis of the indicators below). The patients created veritable discourse concerning themselves and their experience that was like a chapter of their life written by multiple authors. Their synergy led to a request that the professionals take part in the story and this gave rise to the emergence of questions that, setting aside technical aspects, the professionals felt inside themselves.

The patients’ group evolved in various directions:

- *The formation of a group that speaks*: in comparison with the beginning, time and constancy permitted the creation of new relationships and a space in which the participants could exchange experiences;
- *the elaboration of the dissociative process*: participating in the group allowed the patients to become aware of their situation and potential;
- *increased self-narration*: in terms of recounting their experiences before and after the traumatic event;
- *the formation of new key words*: characterizing the experience of the participants.

In relation to the key words, it should first of all be said that there was a transition from the word “disease” to the word “freedom”, in the sense that the condition of disease does not prevent the creation of new strategies for action in everyday life.

In the same way, the word “knowing” was introduced in the sense of being aware of one’s possibilities, with a request for the participation of the professionals encountered during the course of the rehabilitation program. In this way, the group discovered its specific competence and understanding, beginning with a critical awareness of the pathological condition of each of its members and its possible evolution in each case. Being able to speak together broke the taboo.

On an emotive level, the group began by discussing the emotions of “fear” and “shame”, which they all found to be the most intense and pervasive. Confronting these emotions (especially by means of identificative, supportive and sharing interventions) helped the patients to “want to do something” and “want to do something together”.

As bearers of knowledge (also about their diseases and limitations) and sharing (emotive, also in relation to the existence of a limit) led to a wish to share their competence with their families not only with the desire of being listened to and understood, but also with a sense of wanting to introduce their caregivers to a pathway leading to a greater awareness of themselves and their relationships (it is interesting to note that this had been previously proposed by the professionals but had met with refusal, as if the patients’ group needed its own time of elaboration before opening up the possibility of something else).

The creation of the group of professionals was driven by the need to respond to the human question of facing the disease and pain of others as experiences that are common to all human beings. The meetings were centered on sharing the personal emotions triggered by their work in the search for a great culture based on the sharing of their individual experiences, their personal difficulties in facing patients, and their ways of dealing with them. Their being together also allowed them to distinguish between “performance” and “understanding”, in which the former denotes the presence of professional technical skills, and the latter a “taking in” of the other, including other’s experience of life in their own experience.

Reflecting on the experience of the professional led to the emergence of two possible to approaches to a patient:

- as someone other than me, in a dimension of alienness insofar as the other is a bearer of disease (a situation in which technical competence becomes a quasi-omnipotent professional instrument for reading and interpreting a patient’s reality from a distance, also to isolate appropriately from one’s own fears and suffering);
- as someone other like me, in a dimension of alterity because even disease is a shared experience (a situation in which technical competence and the human dimension come together in a rich soil on which to build a relational experience based on being able to approach the emotion of the other as part of the sharing).

The immediate consequences in the two groups were:

- an attempt to create a common language that would help them to face the painful experience of disease;
- an attempt to take the defenses of the professionals into account (being on a more symmetrical plan with a patient means that professional competence truly becomes an addition to the human relationship rather than the basis for distancing oneself from the other);
- the creation of a dynamic and interactive network. Another result from the patient group was the desire emerged of starting a group for the caregivers.

The patients had reflected on the changes affecting them and their repercussions on the members of their families in terms of relationships, roles, time, management and care. The group meets every 3 weeks to discuss practical questions concerning the management of their patient and their time involved, as well as their personal emotional situation. The subjects that have emerged concern their role and their reactions to such a traumatic and unexpected event as that of disease.

### Pilot study

The pilot study considers the evolution of indicators in a sample of 14 patients for a period of 9 months (Table 1 summarizes the sample characteristics).

**Table 1:** Demographic and clinical sample characteristics

Pt	Sex	Age	Pathology	Deficit	Months of disease
AZ	M	20	Cranial trauma TBI	Dysexecutive syndrome; right hemiparesis; left hemisyndrome with ataxia	34
EM	M	66	Right hemispheric stroke	Unilateral spatial neglect; left hemisyndrome	12
PC	M	68	Brainstem hematoma	Ataxia; dysarthria	32
LC	M	69	Parkinson’s disease	Cognitive decline; postural reflex deficit; hypokinetic	70
GI	F	81	Left hemispheric stroke	Right hemiparesis; aphasia	15
SG	F	62	Medullary compression due to malignant dorsal angioma	Flaccid paraparesis	19
LB	M	69	Severe acquired brain injury due to cardiac arrest	Ataxia; memory deficit	8
CB	M	71	Cerebellar stroke	Ataxia; dysarthria; dysphagia	15
AD	F	78	Meningioma; right hemispheric stroke; left femoral fracture	Left hemiparesis paralysis	21
GZ	F	50	Cranial trauma TBI; sub-arachnoid hemorrhage	Unilateral spatial neglect; left hemiparesis	34
RP	F	75	Parkinson’s disease	Hypertonia; tremors	72
AA	F	62	Left hemispheric stroke; right femoral fracture	Right hemiparesis; aphasia	38
AV	M	48	Right hemispheric stroke	Left hemiparesis; frontal syndrome; unilateral spatial neglect	7
MM	F	64	Extrapiramidal progressive syndrome	Ataxia; dysarthria	12

**Fig. 1:**
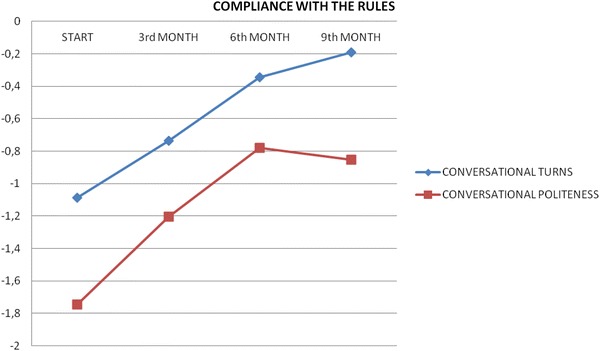
Respect of the conversation rules (turns and politeness)

**Fig. 2:**
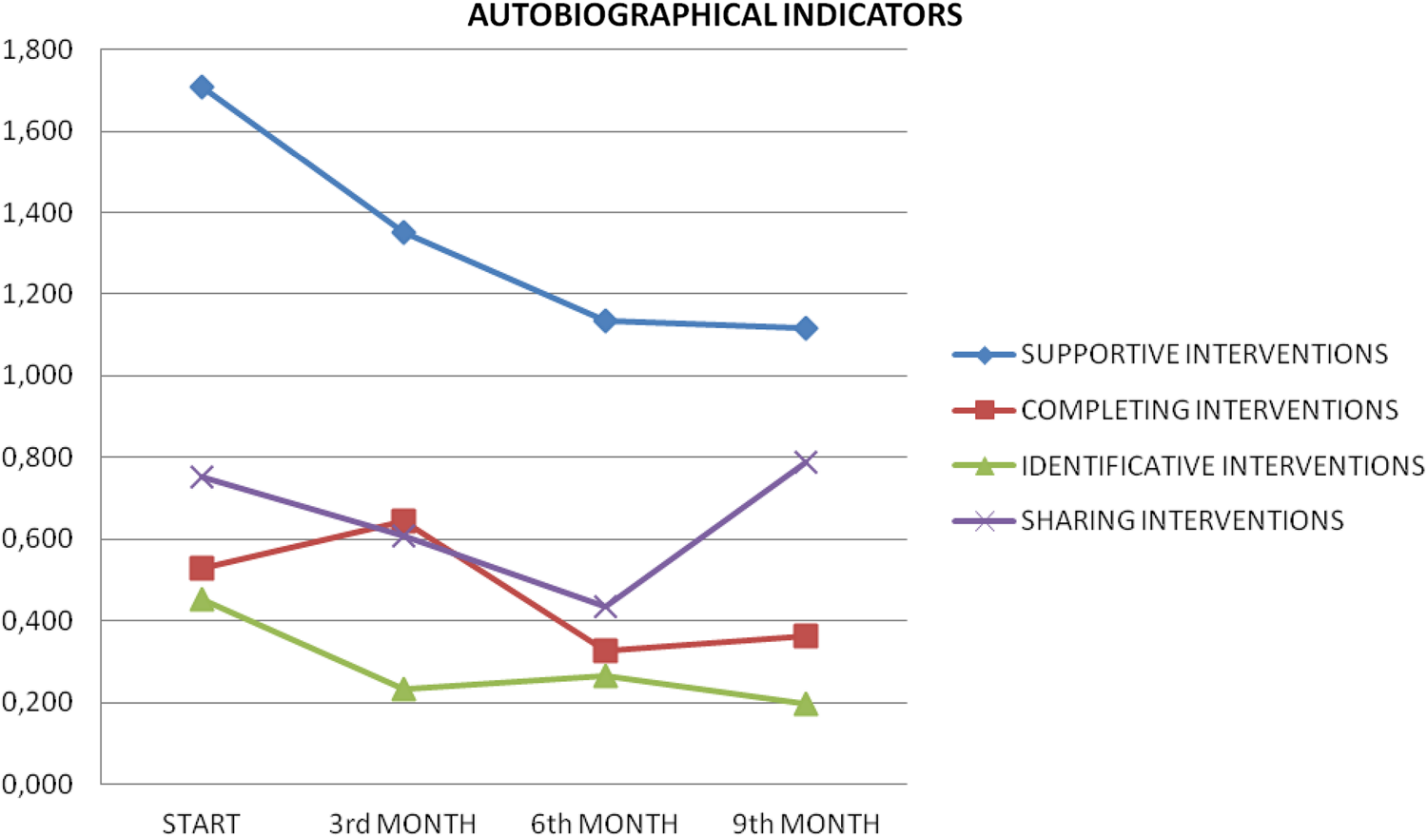
Type of interventions in the conversation meetings

In videotape recording every intervention was marked in line with the described above indicators. Tables 2 and 3 and Figs. 1 and 2 report the mean of recorded interventions in four specific phases: baseline, 3-month follow-up, 6-month follow-up and 9-month follow-up.

**Table 2:** Respect to conversational rules

	Conversational turns	Conversational politeness
Start	−1.086	−1.746
3rd month	−0.735	−1.205
6th month	−0.345	−0.781
9th month	−0.191	−0.853

**Table 3:** Type of interventions in the conversation meetings

	Supportive interventions	Completing interventions	Identificative interventions	Sharing interventions
Start	1.709	0.528	0.452	0.753
3rd month	1.350	0.645	0.231	0.608
6th month	1.134	0.327	0.267	0.436
9th month	1.116	0.363	0.197	0.788

Every infraction to the “conversationalism rules” was recorded. As shown in Fig. 1, infractions were progressively reduced in frequency, with better conversation fluency and communication roundness. Group members learned an “active listening position”; through the silence, it is possible to confront with other people that share the same experience.

The prevalence of supportive interventions was reduced while elements of sharing progressively increased. The progressive reduction of identification and accomplish could lead to a progressive increased consciousness of self and of their disorders, and to an increased disease consciousness and a better confront capability with other than self who share the same experience.

## Discussion

Our experience of group meetings involving patients with severe brain damage of various kinds supports the affirmation of Paul Watzlawick [13] that “it is not possible not to communicate”.

### The evolution of the group

First of all, the meetings showed the presence of extra-verbal communication, which led to what Lai [7] called “happy conversation”: i.e., the pleasure of being together with others and the perception that it is possible to participate in an inter-human relationship regardless of the cognitive and communicative capacities that establish its form and content. This made the people happy and pleased there and, subsequently, led them to bring into play their personal condition of which they had become more critically aware.

This non-verbal communication was accompanied by a change in content which, in both the patients’ and the professionals’ groups, led to the emergence of a need to pass from “performance” to “comprehension”, in the sense of “taking the whole”. This should help understood the change of a “technical” profession centered on executing a task (taking care of patients’ illness) toward a profession that also contemplates “taking in” the other and his/her situation. It became immediately clear that this need concerned the professionals more than the patients. The technical competences tend to lead to a response based on personal knowledge that loses sight of the other and exposes oneself to the greatest frustrations: there is no answer, or at least it is not one based on manuals and standard operating procedures.

Recognition of this also led to the recognition of a shared pathway consisting of reciprocal questions and reciprocal searches for the answers, not with the presumption that these are the right questions, but with the certainty that they are the truest.

In substance, it was a case of passing from “What do I do?” to “Who am I?”

The most significant transition occurred when it became clear that the disease had demonstrated that it was a question that made sense.

It was together understood that one could and should take a step backwards when faced with the pain of the other and avoid forced activism. In this way, it is possible to discover a silence that allows the other to make himself present and discover inside in oneself the freedom and desire to look at the other as he/she is, and to share this look with the other, thus experiencing the relaunching of oneself in the face of one’s own “I am”, and knowing that it is simultaneously experienced by the other.

The question therefore becomes not what but who is being treated.

The sharing of the experience that arises after humble acceptance of the fact that reality imposes itself was extraordinary.

The last elements to emerge were:

- the function of beauty
- the new dimension of time.

The experience unmasked the need to reach the point to join “disability” and “desire”. This passage is possible through the initial painful awareness of one’s own condition (awaited in particular by the professionals), followed by the sharing of one’s condition (as part of the phenomenon we have called identification/projection). By means of sharing the betrayal of the body and the subsequent experience of affective communication (the happy conversation), it is possible to arrive at the expression of desire and expectation.

### Some considerations concerning language and communication

Mankind has an innate (ontological) need for “meaning”, a vocation for “making sense” that Martin Heidegger [14] sees in the “intimate signifying of existing”.

Therefore, whenever human beings look at the world, they place a sign (signum facere) in a dramatic, incessant and strenuous attempt to overcome their feeling of “extraneousness” and “disposability” in the “inauthenticity of the world”. They perceive a detachment from a constitutive part of themselves, a castaway in an unknown world, tossed by a perilous sea that may carry them “to know” before than “to learn to understand”.

The process of “knowledge” in this sense carries with it the detachment of the subject–observer from the object/fact-observes by means of the coded mediation of symbols (logic-discursive categorizing knowledge). It is precisely this distance between the subject and the object that permits knowledge. This detachment is perceived as solitude (depression), which ontologically underlies the way in which human beings (the only point in nature in which they become aware of themselves) exist in the world. They have to find the capacity to confront the world within themselves.

It can therefore be hypothesized that there is a transition from (more indefinite) signs to (more stable) symbols as vehicles of the unfolding of the relationship between subject and object.

If we observe the logical contraposition of subject and object with phenomenological eyes, as co-agents in the fact-finding act, they do not seem to be as distinct and finite as they appear in our common fact-finding experience, but become a Biswangerian modality [15] of “being human with human beings”, a “subject body” insofar as it is “placed in front” a world in its being (Da sein), and “in front of humans” in its “novelty” (die Wirheit) [14]. This implies an essential and reciprocal ontological belonging of “I” and “you” by means of which (with reference to the dialogical principle of Martin Buber [16]), being is founded on its capacity of maintaining a close relationship with the other.

The tie with the other than self therefore becomes the possibility of knowing, moving, existing, and defining the self that is otherwise ineluctably submerged in the shipwreck of birth and detachment. This is why man is defined as I in action, why it is necessary to start with oneself when setting out to discover the real, when the I-in-action must be at the center [14].

It is therefore clear when we deal with signs and consider a sign as an expression of the instance of man as signifier, we admit that fact-finding act takes place at the moment in which we give something a name, as in the Book of Genesis.

Furthermore, as pointed out by Minkowski [17], a sign allows a space between the signified and the signifier. In the words of Marcel Foucault [18], it is necessary to recognize “… an excess of signified over signifier, a residue that is necessarily not formulated by a thought that language has left in the shade, a residue that is its very essence”.

In this sense, in addition to its extraordinarily value as a socially shared symbolic construct that allows reality to be dominated, language also acquires the importance of being a semantic symbol, a set of signs oriented toward establishing, maintaining and enriching the relationship with the other.

This makes it possible to see the symptoms of dementia and other forms of cerebral damage in a new light: echolalia, confabulation, repetitive questions, bizarre gestures and perplexed smiles all reflect the strenuous efforts of someone who, betrayed by his brain and deprived of the instruments that would allow him to interact easily with the world, does not want to be detached from the object precisely because, without this contact, his condition is that of someone who has been cast defenseless into solitude.

There is therefore a clear implicit intention of not wanting to be overwhelmed by the abstract tempest of symbols but wanting to abandon an environment in which a subject observes an object with the judgemental categorizing detachment of conventional standards (psychometric tests, diagnostic investigations, history, etc.), and open up a panorama in which the fact-finding takes place in the unfolding condition of “being human with human beings”, in the fundamental alterity of existential encounter.

A word itself is therefore not an exact cipher codified in an inter-individual relationship, but develops with it as a sign of the phenomena associated with its continuation.

### Limitations

The first is the fact that this is simply an account of what was observed, devoid of any specific measurements. A future study should be designed on the basis of more rigorous measurements.

The second is that the study did not consider the effects of the meetings on the efficacy or duration of rehabilitation treatment. This seems to be particularly important because psychiatric or psychological factors [2] or elements of faith may significantly influence prognosis (for example of a tumor). A comparison between the conversationalism group and a control group doing a “conventional rehabilitation program” may have helped therapists to rate the program efficacy.

Further consideration might be given to the fact that the members entered and left the group depending on their rehabilitation program. It would be interesting to discuss the experience with the participants to verify whether they acknowledge and agree with the observed changes. A patients’ feedback could help therapists to know if patients are really “happy”, “pleased” and critically aware of their condition at the end of the program.

### Future perspectives

First of all, during the course of their own meetings, the patients asked that their caregivers be given a space in which they could meet to discuss the dynamics triggered by the traumatic event and the resulting disability. The creation of a space in which family members reflect on the traumatic event and exchange their thoughts could allow them their own time of elaboration (also in relation to the frustrations related to their new roles) and could lead to the creation of new dynamics aimed at reinforcing their coping strategies and broadening their vision of possible solutions that could be used in particular situations (managing their free time, managing the exercises involved in the rehabilitation program, etc.). A recent review confirmed this need, underlying the importance of dyadic interventions for people with cognitive impairment and their caregivers on mutual understanding and communication to partners’ well-being and relationship quality within the caregiving process [19].

Second, there is no doubt that the experience needs to be validated by means of indicators. Furthermore, it would be worth investigating whether it had a concrete effect on rehabilitation outcomes, and if so, the significance of this effect.

At last, another step could be to look at significant parts of the video-recordings together with the participants to have their feedback.

## Conclusions

The described experience once again poses two new essential questions: that of the climate which, as pointed out by Benedetto Saraceno [20], seems to be unmeasurable indicator, and that of reality, as written by Etti Hillesum [21].

Another important consideration is that even in patients with severe communication deficit, like in Alzheimer disease or intellectual disability, the communication skills may improve [6, 8, 9].

One last reflection is that the experiences made it possible to join the two apparently unconnected words of desire and disability. What emerged during moments of the conversations was the reawakening of a profound desire that is rooted in human nature itself and concerns being here regardless of one’s limitations.

One certain point constructed within the groups is that the word “acceptance” is not useful in relation to this experience or the process of rehabilitation. This is important because it reflects a wish for continuous improvement. Furthermore, the word “acceptor” seems to represent a point of arrival and the beginning of a situation of involution that contrasts with the natural tendency to human beings to evolve.

As emerged from the patients’ group, the “disability of today” that they experience is also colored by positive aspects, such as having more time or being able to look at previously unseen family dynamics. Without wishing to glorify the condition of disease, this represents what can be defined as the beauty of human limitations and the continuous desire to go beyond them.

We believe that this experience has helped mental healthcare professionals to focus their attention to the “person” instead of the illness. Their knowledge can become a tool to welcome patients’ demands and not just the goal of their job. In patients we observed an “emotional rise”, through a change in other sight on them.
